# “THE HARDEST JOB IS A HOSPITAL DIRECTOR”: LEADERSHIP PERSPECTIVES PRIORITIZING RURAL GERIATRICS IN THE VA

**DOI:** 10.1093/geroni/igae098.0979

**Published:** 2024-12-31

**Authors:** Lynette Kelley, Emily Franzosa, Eileen Dryden, Lauren Moo, Lauren Hall, William Hung

**Affiliations:** Eastern Colorado Health Care System Rocky Mountain Regional VA, Aurora, Colorado, United States; James J Peters VA Medical Center, Bronx, New York, United States; Veterans Health Administration, Bedford, Massachusetts, United States; Department of Veterans Affairs, Bedford, Massachusetts, United States; James J Peters VA Medical Center, Bronx, New York, United States; James J Peters VA Medical Center, James J Peters VA Medical Center, New York, United States

## Abstract

The Veterans Health Administration, like other health systems, faces the challenge of meeting older rural Veterans’ needs. VA leaders must balance these complex needs with other local priorities, and, at the same time, a large number of national directives. Little is known about what influences the process of setting priorities and making decisions from the standpoint of healthcare leaders. We aimed to better understand how healthcare leaders weigh priorities of the health system and how their processes in turn impact the prioritization of services. We interviewed 11 recently retired local, regional and national health system leaders about their experience and how priorities in health systems can be aligned to improve the quality and reach of rural geriatric care. All leaders agreed that the highest priority is always Veteran access to high-quality care (national mission) but noted differences in what that might look like within their own settings (e.g., prioritizing the expansion of certain specialty care or clinics depending on the population). Leaders described how they weighed multiple competing priorities to decide what was of highest urgency to serve their local Veteran population while balancing national directives. Local leaders described juggling multiple administrative, clinical and leadership responsibilities daily; regional and national leaders in oversight positions agreed that the complex interplay between national and local needs may leave local leaders feeling overwhelmed. We map our real-world data to standard leadership priority frameworks, highlighting gaps between theory and practice to identify effective and practical leadership strategies.

